# Existence of Th22 in children and evaluation of IL-22 + CD4 + T, Th17, and other T cell effector subsets from healthy children compared to adults

**DOI:** 10.1186/s12865-016-0158-8

**Published:** 2016-06-23

**Authors:** Erxia Shen, Mengjie Wang, Hairui Xie, Ruqiong Zou, Qiwen Lin, Lili lai, Fujun Li, Zhimei Liang, Yanran Xu, Maohua Zhou

**Affiliations:** Department of Pathogenic Biology and Immunology, Sino-French Hoffmann Institute, School of Basic Science, Guangzhou Medical University, Guangzhou, 510182 China; Department of Cancer Immunology and Virology, Dana-Farber Cancer Institute, Boston, MA 02115 USA; Yuexiu District Children’s Hospital of Guangzhou, Guangzhou, 510115 China; Guangzhou Blood Center, Guangzhou, 510095 China; Department of Laboratory Medicine, Guangdong General Hospital, Academy of Medical Sciences, Guangzhou, 510080 China

**Keywords:** Interleukin-22, Children, Adults, Memory T cells, CD4 T helper

## Abstract

**Background:**

Children are prone to get infections, especially in the respiratory system and the gut mainly because their immune system is immature. T cells significantly contribute to the prevention of infections, and different helper T cell (Th) subsets play different anti-pathogen roles. Interleukin (IL)-22 producing by T-helper 22 cells (Th22) play an important role in host defense against Gram-negative bacterial organisms in gut and lung. T-helper 17 cells (Th17) protect against extracelluar bacteria and fungi especially at the epithelial surface. However, there is no report comparing IL-22 producing T cells and Th17 cells in healthy young children to adults.

**Methods:**

Flow cytometry (FCM) was used to observe whether Th22 subset existed in the peripheral blood of healthy young children. Meanwhile, we determined the frequencies of Th subsets including Th17, Th1 and Th2, cytotoxic T (Tc)1 subset, CD4+ and CD8+ memory T cells in the peripheral blood of both young children and adults.

**Results:**

In the present study, we demonstrated that Th22 subset existed in peripheral blood of children, with IL-22 mainly secreted by CD4 + CD45RO+ memory T cells. Moreover, we observed that IL-22 + CD4 + T cells and Th subsets including Th17, Th1, and Th2 frequencies of young children (1–6 years old) were significantly lower than adults. While the Th1 frequency from Group A (1–3 years old) was markedly lower than that from Group B (4–6 years old). No significant differences of Th17 or IL-22 + CD4 + T cells frequencies were observed between these two groups. In addition, Tc1 subset frequencies were also remarkably lower in young children than in adults. Furthermore, lower frequencies of CD45RO+ memory CD4+ and CD8+ T cells in young children than in adults, and significant correlation between CD45RO+ memory CD4 + T cells and IL-22 + CD4 + T cells, Th1, Th17 were observed.

**Conclusions:**

Th22 subset exists in the peripheral blood of young children. Compared with adults, there are lower frequencies of IL-22 + CD4 + T cells, as well as Th1, Th17, Th2 and Tc1 subsets in the peripheral blood of young children.

## Background

Significant phenotypic differences between T cells of neonates, infants and children have been reported, suggesting a gradual development of cell-mediated immunological defense mechanisms [1, 2]. During infancy and early childhood, the immune system is immature. However, exposure to pathogens and vaccination antigens stimulate a battery of activation and maturation processes, which allow for acquired immune memory. The gradual development of a fully mature immune system is a major reason why neonates, infants and young children are more prone to get infectious diseases, such as respiratory tract infections, compared to adults [3, 4].

T cells significantly contribute to pathogen-specific adaptive immune responses and protect against infection from a broad and different species of potential pathogens. In response to antigen stimulation, naïve CD4 + T cells activate, proliferate, and differentiate into distinct effector CD4+ T helper (Th) cell lineages in antigen specific ways. The process of Th cell differentiation is intricately regulated, and different effector lineages have different functions. There are at least four Th subsets: Th1, Th2, Th17 and Th22. Th1 cells produce the effector cytokines interferon gamma (IFN-γ) and Interleukin (IL)-2 to protect from intracellular pathogens, while Th2 cells secrete Interleukin (IL)-4, Interleukin (IL)-5 and other effector cytokines involved in clearance of helminthes [5–7]. IL-17 secreting Th17 cells protect against extracelluar bacteria and fungi especially at the epithelial surface [8–10]. And Th22 subset is the most recently identified Th subset. This subset produces the lineage-defining cytokine IL-22, in the absence of IFN-γ and IL-17 secretion or expression of Th1- and Th17-associated transcription factors T-bet and retinoid acid-related orphan receptor gammat (RORγt) respectively. The Th22 associated transcription factor is a Aryl hydrocarbon receptor (AHR) which is important for expression of IL-22 [11, 12]. IL-22 functions to sustain the integrity and barrier of mucosal epithelial tissues and plays an important role in host defense against Gram-negative bacterial organisms (particularly in gut and lung) [13–15]. Normally, all of the different Th effector subsets work together to protect against infection induced by pathogens.

To our knowledge, there is no report which compares the levels of IL-22 producing T cells and Th17 cells in healthy young children to adults. In the present study, we firstly confirmed that Th22 subset existed in the peripheral blood of young children, and evaluated the phenotype of this subset. Then, we found that IL-22 + CD4 + T cell frequencies of young children were significantly lower than adults, as well as Th1, Th2, Th17, and Tc1 frequencies. Furthermore, the level of Th1 frequency from Group A (1–3 years old) was markedly lower than from Group B (4–6 years old). However, there were no significant differences of levels of Th17 and IL-22 + CD4 + T cell frequencies between these two groups. We demonstrated that there were higher frequencies of CD45RO+ memory CD4+ or CD8+ T cells in adults than in young children. Significant correlation between CD45RO+ memory CD4 + T cells and IL-22 + CD4 + T cells, Th1, Th17 was observed. Increased exposure, over a lifetime, to antigens may explain in part why young children are prone to infectious diseases.

## Methods

### Subjects

Twenty healthy young children (aged between 1 and 6 years old, 10 girls and 10 boys) were recruited from Yuexiu District Children’s Hospital of Guangzhou and 3 ml blood samples were collected. Children enrolled in the study received formula, medical checks and got schedule vaccination according to Planned Immunization Program of China. These individuals had no acute or chronic infectious diseases, nor any clinically significant diseases or findings in the medical history that might compromise the immune function (e.g., diabetes mellitus, asthma, rheumatoid arthritis, and tumors). Twenty-three healthy adult volunteers were recruited from Guangzhou Medical University, and they donated blood samples for this study. The mean age of healthy volunteers (11 males and 12 females) was 26 years of the age (range 19–40 years old). These adult individuals had no acute or chronic infectious diseases, autoimmune diseases or tumors. Written informed consent was obtained from individual participants or their parent (for young children). The protocol of this study was established according to the guidelines of the 1975 Declaration of Helsinki and approved by the Ethics Committee of Guangzhou Medical University (Guangzhou, China) and the Ethics Committee of Yuexiu District Children’s Hospital of Guangzhou (Guangzhou, China).

### Reagents

Anti-CD3 FITC, anti-CD3 APC, anti-CD4 PerCP-cy5.5, anti-CD8-PerCP-cy5.5, anti-CD8 FITC, anti-CD45RO FITC, anti-CD45RO PE, anti-IFN-γ FITC, anti-IFN-γ APC, anti-IL-4 PE and isotype-matched control mAbs were purchased from BD PharMingen (San Diego, CA, USA). Anti-IL-22 PE was purchased from R&D Systems (Abingdon, UK). Anti-IL-17 APC was purchased from eBioscience (San Diego, CA, USA). Phorbol myristate acetate (PMA), ionomycin, saponin and Brefeldin A (BFA) were purchased from Sigma-Aldrich (Fluka, Sigma, USA).

### Cell isolation

3 ml heparinized blood by venipuncture of donors were harvested. Then the blood was transported to our laboratory on ice in 4 h. Peripheral blood mononuclear cells (PBMCs) were isolated from using Ficoll-Hypaque density gradient centrifugation, and washed twice in Hank’s solution. These cells were finally adjusted to a final concentration of 2 × 10^6^ /ml in complete Roswell Park Memorial Institute (RPMI) 1640 medium (GIBCO, Grand Island, NY, USA) supplemented with 10 % Fetal calf serum (FCS) (Sijiqing, China), 50 mM 2-mercaptoethanol, 100 U/mL penicillin, 100 mg/mL streptomycin and 2 mM L-glutamine (all from GIBCO).

### Cellular staining and flow cytometry

For intracellular staining, PBMCs were stimulated with PMA (20 ng/mL) and ionomycin (1 μg/mL) for 4–6 h at 37 °C in a 5 % CO_2_ humidified atmosphere. Brefeldin A (BFA, 10 μg/ml) was added into the culture at the end of first hour during the incubation. The cells were collected, washed twice in cold phosphate buffered-saline (PBS), cell surface staining Abs were added and incubated at 4 °C for 25–30 min. The cells were washed twice and fixed with 4 % paraformaldehyde and re-suspended in permeabilization buffer (PBS containing 0.1 % saponin and 0.5 % BSA). After incubation at 4 °C for at least 2 h, intracellular cytokine staining Abs and FMO control were added and incubated at 4 °C for 25–30 min. Cells were washed with PBS, resuspended in cold staining buffer, determined by BD FACSCalibur Flow Cytometer (BD Biosciences, San Jose, CA). For Fig. 6, only the cell surface staining without stimulation was done. Lymphocytes were gated on forward and side scatter profiles and analyzed using FlowJo software (Treestar, San Carlos, CA, USA).

### Statistical analysis

Comparison between two groups was performed by unpaired Student’s *t*-test for two tails. To evaluate correlation, Pearson’s correlation coefficients were used. *p* value of < 0.05 was considered to be statistically significant. All statistical analyses were performed using GraphPad Prism (version 5.0 Software Inc, San Diego, CA, USA).

## Results

### Th22 subset exists in blood of healthy young children

**Fig. 1:**
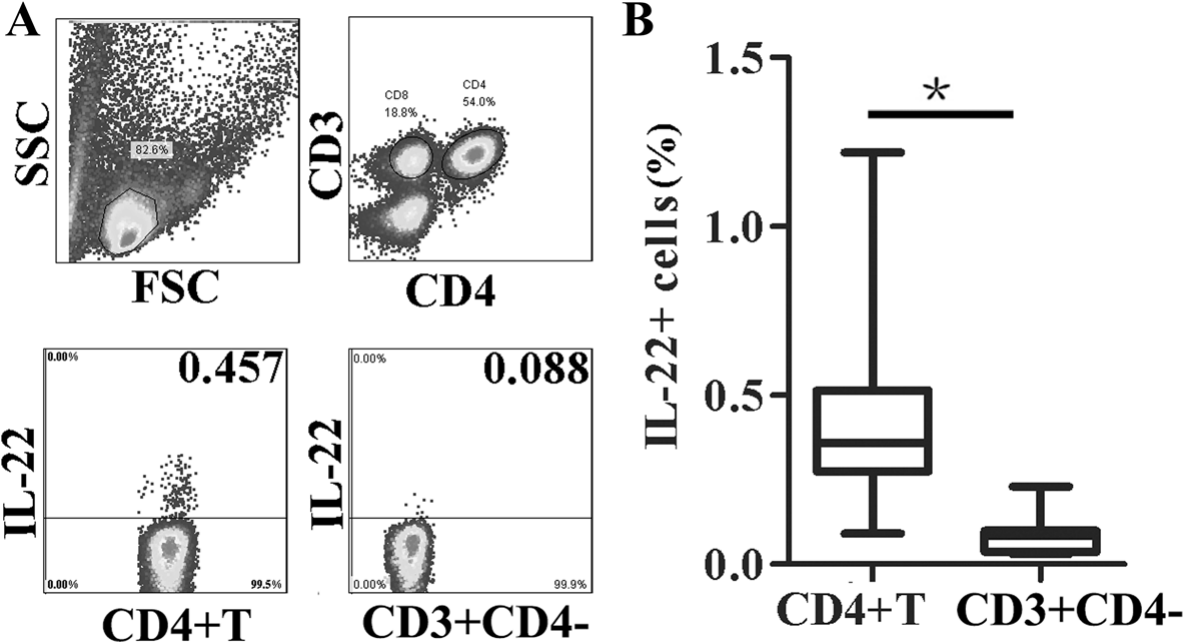
IL-22 is produced by CD4 + T cells in healthy young children. PBMCs from healthy young children were prepared and cultured with PMA and ionomycin for 4–6 h, then cells were harvested, fixed, permeabilized, and cell surface and intracellular staining were done and determined by FACS. **a** IL-22 production by CD4 + T or CD3 + CD4-T cells from one representative donor was shown. Lymphocytes were first gated on CD3 + CD4 + (CD4 + T cells) or CD3 + CD4- cells and then analyzed for IL-22 production. **b** The statistical data results of the percentages of IL-22+ cells in CD4 + T cells or CD3 + CD4-T cells were shown (*n* = 9). The median is represented by horizontal line, the interquartile range by box and the minimum to maximum range by whiskers. *, *p* < 0.05

**Fig. 2:**
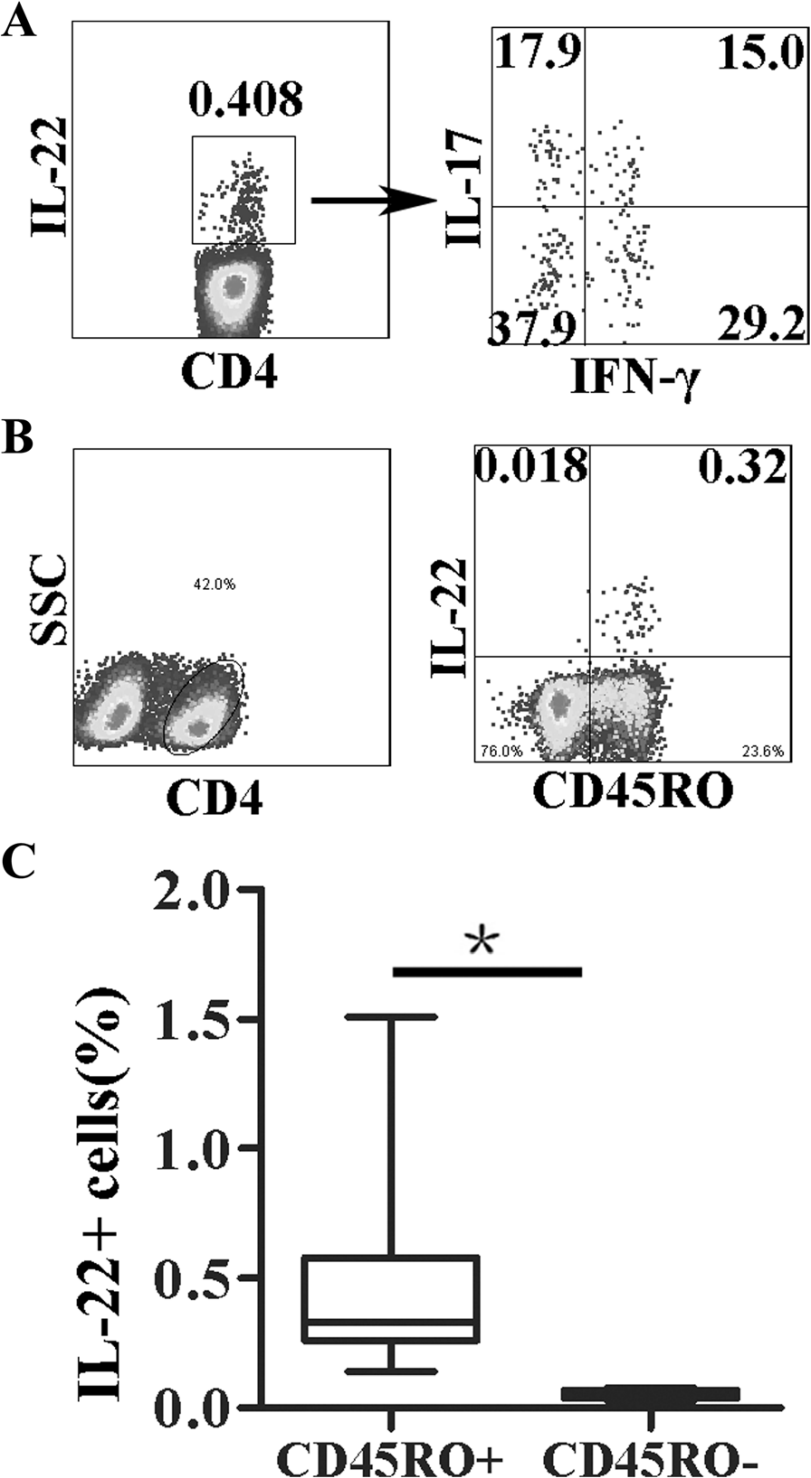
Th22 cells exist in blood of young children and are memory CD4 + T cells. PBMCs from young children were stimulated with PMA and ionomycin for 4–6 h. Cell surface and intracellular staining was determined by FACS. **a** IL-22 + CD4 + T cells, IL-17 and IFN-γ production by IL-22 + CD4 + T cells from one representative donor were shown. CD4+ cells were gated from lymphocytes, and IL-22 producing CD4 + T cells were analyzed for IL-17 and IFN-γ production. **b** IL-22 production by CD45RO+ memory CD4 + T cells was shown from one representative donor. CD4 + T cells were gated from lymphocytes, and IL-22 and CD45RO expression was analyzed. **c** The statistical results of the percentages of IL-22 + cells by CD45RO+ or CD45RO- CD4 + T cells were shown (*n* = 7). The median is represented by horizontal line, the interquartile range by box and the minimum to maximum range by whiskers. *, *p* < 0.05

We first determined whether peripheral T cells from young children could produce IL-22. As shown in Fig. 1a, 0.457 % of CD4 + T cells and 0.088 % of CD3 + CD4- T (majority of them are CD8 + T) cells produced IL-22 (from one representative result) in young children. Statistical results (Fig. 1b) showed that the frequency of IL-22 produced by CD4 + T cells was significantly higher than that by CD3 + CD4- T cells (*p* < 0.05). This finding indicated that CD4 + T cells were the majority cells producing IL-22 not CD8 + T cells. As shown in Fig. 2a, 37.9 % of IL-22 + CD4 + T cells produced neither IFN-γ nor IL-17 and were therefore considered Th22 cells (IL-17-IFN-γ-IL-22 + CD4 + T cells). Further characterization of the IL-22 producing CD4 + T cells showed a memory phenotype that 0.32 % of CD4 + T cells produced IL-22 and were CD45RO positive, but only 0.018 % of CD4 + T cells produced IL-22 and were CD45RO negative. (Fig. 2b). Statistical results demonstrated that the frequency of IL-22+ in CD45RO + CD4 + T cells was markedly higher than that of IL-22 expression in CD45RO-CD4 + T cells (Fig. 2c). The above results demonstrated that Th22 subset existed in peripheral blood of healthy young children, and majority of this subset cells were CD45RO+ memory T cells.

### Comparison frequencies of IL-22 + CD4 + T cells, Th17 or Th1 cells from young children with adults

**Fig. 3:**
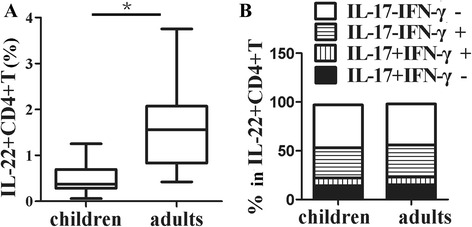
Lower frequencies of IL-22 + CD4 + T cells from young children compared to adults. **a** the statistical results of frequencies of IL-22 + cells by CD4 + T cells from young children (*n* = 20) compared with adults (*n* = 23) were shown. **b** the statistical results of frequencies of IL-17-IFN-γ-, IL-17-IFN-γ+, IL-17 + IFN-γ + and IL-17 + IFN-γ- cells by IL-22 + CD4 + T cells from PBMCs of young children compared with adults were shown. The median is represented by horizontal line, the interquartile range by box and the minimum to maximum range by whiskers. *, *p* < 0.05

We compared frequencies of IL-22 + CD4 + T cells from young children with adults. As shown in Fig. 3a, the proportion of IL-22 + CD4 + T cells from young children were significantly lower than those from adults (*p* < 0.05). Furthermore, we compared four subsets including IL-17-IFN-γ-, IL-17 + IFN-γ-, IL-17 + IFN-γ+, IL-17-IFN-γ + cells in IL-22 + CD4 + T cells from children and adults, respectively. No statistical significance was observed in any of these IL-22 + CD4 + T cells subpopulations between young children and adults (Fig. 3b).

**Fig. 4:**
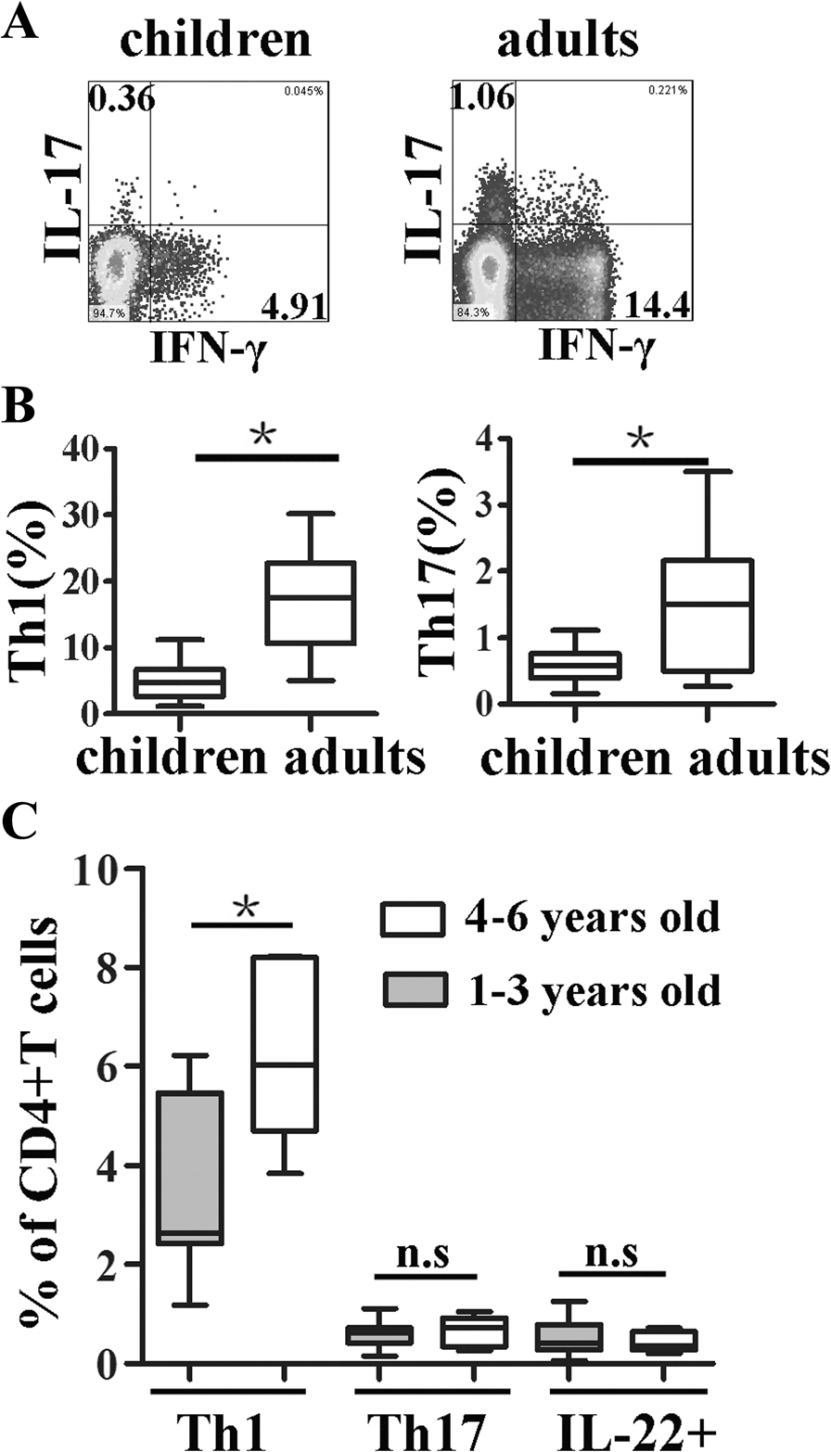
Comparison of Th1, Th17 and IL-22 + CD4 + T cells frequencies from age striated children groups and adults. **a** one representative result of frequencies of Th1 and Th17 cells in CD4 + T cells both from healthy young children and adults by FACS was shown. **b** the statistical results of Th1 and Th17 cells in CD4 + T cells were shown (children: *n* = 20; adults: *n* = 23). **c** Frequencies of Th1, Th17 and IL-22 + CD4 + T cells from age 1–3 years old (Group A, *n* = 10) and 4–6 years old (Group B, *n* = 10). The median is represented by horizontal line, the interquartile range by box and minimum to maximum range by whiskers. *, *p* < 0.05. n.s, no significant difference

Concurrently, we determined Th17 and Th1 frequency from both young children and adults. The results showed that there were 0.36 % Th17 cells and 4.91 % Th1 cells (from one representative result) in young children, and 1.06 % Th17 cells and 14.4 % Th1 cells (from one representative result) in adults (Fig. 4a). Frequencies of Th1 or Th17 cells from young children were markedly lower than those from adults (Fig. 4b, *p* < 0.05). Moreover, when young children were divided into 2 age groups 1–3 years old (Group A) and 4–6 years old (Group B) we found that the Th1 frequency from Group A was markedly lower than from Group B (*p* < 0.05). However, there were no significant differences of levels of Th17 and IL-22 + CD4 + T cells frequencies between these two groups (Fig. 4c, *p* > 0.05). Our findings indicated that IL-22 + CD4 + T cell, Th subsets including Th17 and Th1 frequencies of young children were significantly lower than adults.

### Lower percentages of Th2 and Tc1 subsets were observed in young children compared with adults

**Fig. 5:**
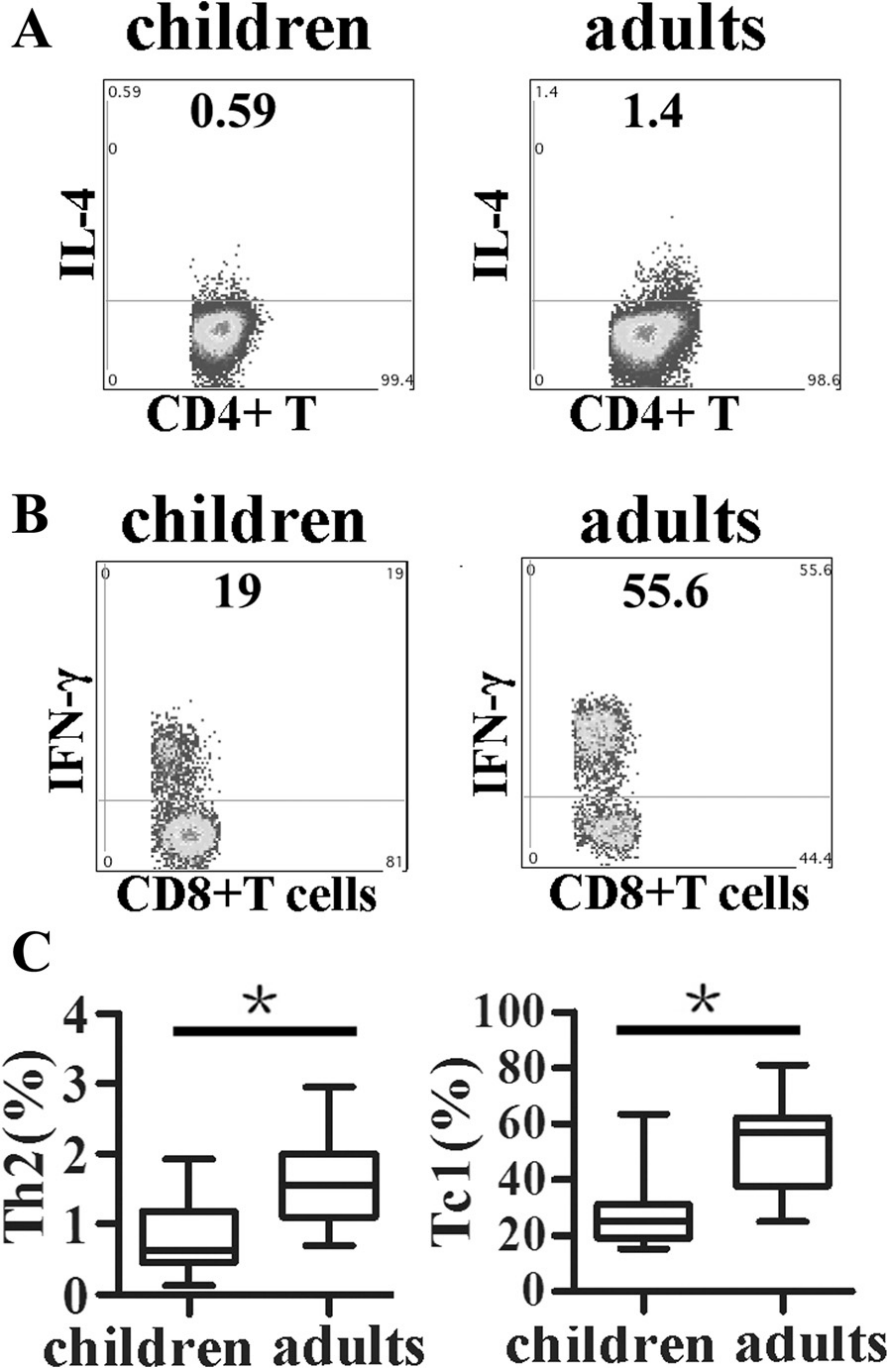
Lower levels of frequencies of Th2 and Tc1 cells from young children compared to adults. **a** one representative result of frequencies of Th2 (gated on CD3 + CD4+) from young children or adults by FACS. **b** one representative result of frequencies of Tc1 (gated on CD3 + CD8+) from young children or adults by FACS. **c** compared with the percentages of Th2 and Tc1 cells from young children and adults (children: *n* = 20; adults: *n* = 23). The median is represented by horizontal line, the interquartile range by box and minimum to maximum range by whiskers. *, *p* < 0.05

We compared frequencies of Th2 and Tc1 cells (IFN-γ + CD8 + T) from young children and adults. The results showed that there was 0.59 % Th2 cells in young children and 1.4 % Th2 cells in adults (from one representative result) (Fig. 5a). Frequency of Tc1 cells from one representative result of young children or adults was 19 and 55.6 %, respectively (Fig. 5b). Statistical results demonstrated that levels of Th2 or Tc1 cells proportions from young children were significantly lower than those from adults (Fig. 5c, *p* < 0.05).

### Correlation of Th1, Th17 and IL-22 + CD4 + T cells with CD45RO+ memory CD4 + T cells

**Fig. 6:**
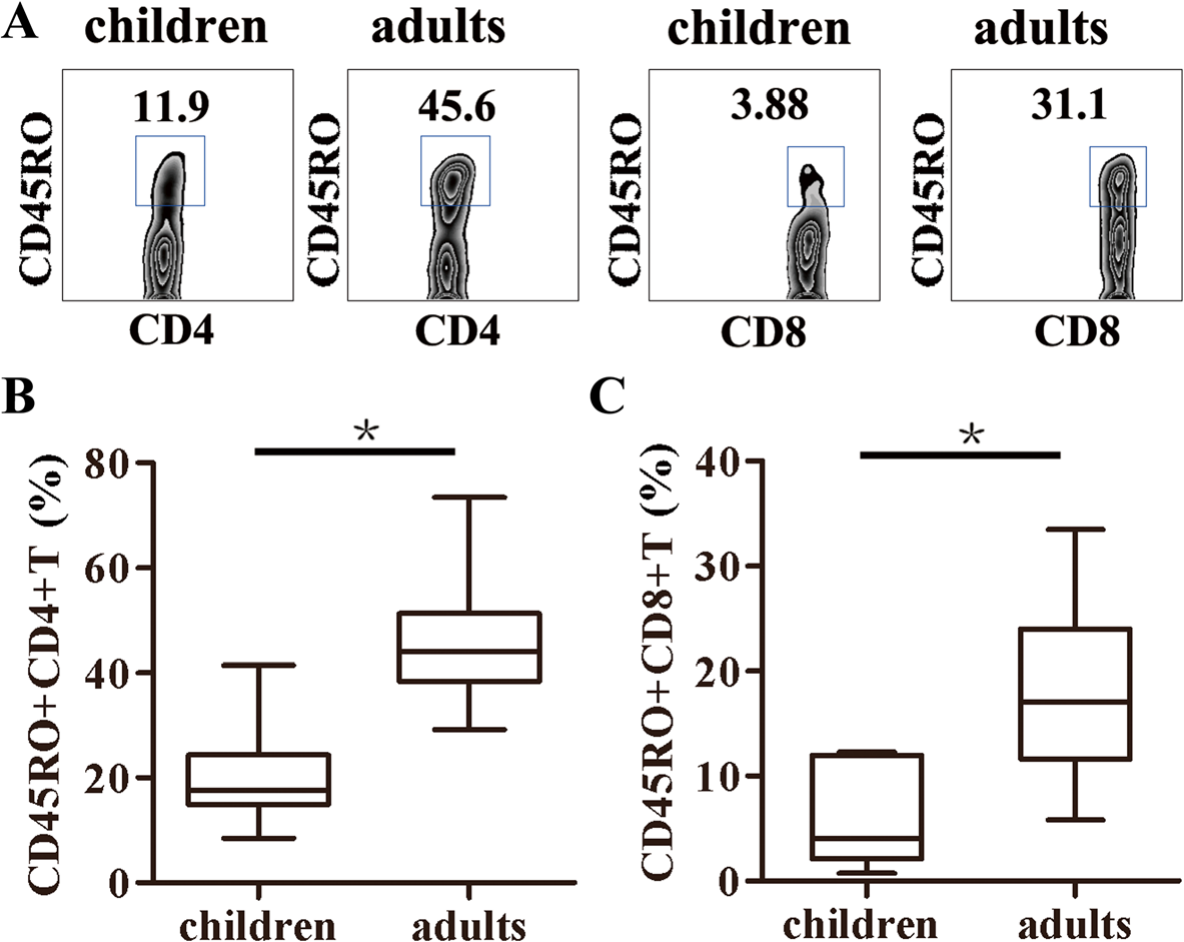
Comparison of frequencies of CD45RO + memory CD4+ or CD8 + T cells from young children compared to adults. CD45RO expression on CD4+ or CD8 + T cells was analyzed. **a** one representative result of CD45RO on CD4 + T (gated on CD3 + CD4+) or CD8 + T (gated on CD3 + CD8+) from young children or adults by FACS. The statistical results of frequencies of CD45RO + cells in CD4+ (**b**) or CD8 + T cells (**c**) from young children compared with adults were shown (children: *n* = 16; adults: *n* = 21). The median is represented by horizontal line, the interquartile range by box and minimum to maximum range by whiskers. *, *p* < 0.05

**Fig. 7:**
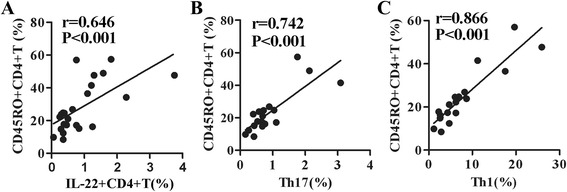
Correlations of IL-22, IFN-γ, IL-17 production with CD45RO expression by CD4 + T cells. Correlations of the frequencies of IL-22 + CD4 + T cells (**a**), the frequencies of Th1 cells (**b**), and the frequencies of Th17 cells (**b**) with the frequencies of CD45RO + CD4 + T memory cells. Correlations were assessed using the spearman correlation coefficient

As naive T cells do not generally secrete IL-22, IL-17, IFN-γ or IL-4, flow cytometric analysis of circulating levels of IL-22 + CD4 + T cells, Th17 and other T cell subpopulations might reflect differential levels of antigen-driven T memory cells as the children immune system matures. Therefore, we determined and compared the frequencies of CD45RO + CD4+ and CD45RO + CD8+ memory T cells of PBMCs from both of these two groups. The results showed that the percentages of CD45RO+ memory CD4 + T cells or CD45RO+ memory CD8 + T cells from children group were significantly lower than those from adult group (Fig. 6, *p* < 0.05). Moreover, a significant correlation (*r* ≥ 0.6) was demonstrated between CD45RO+ memory T cells and IL-22 + CD4 + T cells, Th1, Th17, respectively (Fig. 7a, b and c).

## Discussion

In the present study, we stimulated T cells with PMA and inomycin to induce a non-specific activation of T cell cytokine production, and assessed the overall functional capacity of T cells from both young children and adults. We demonstrated that Th22 subset existed in the peripheral blood of young children, and IL-22 was mainly produced by CD4 + CD45RO+ memory T cells. Compared with adults, there was lower frequency of IL-22 + CD4 + T cells in the peripheral blood. Then we assessed other Th subsets including Th1, Th17, Th2, and Tc1 subsets from both young children and adults. The results showed that young children had lower frequencies of Th1, Th17, Th2 and Tc1 subsets than adults. Furthermore, there were lower percentages of memory CD4+ or CD8+ T cells in young children than in adults. Significant correlation between CD45RO+ memory CD4 + T cells and IL-22 + CD4 + T cells, Th1, Th17 was demonstrated. Lower frequencies of Th or Tc1 effector subsets in young children might partially explain why young children are prone to get infections.

At first, we demonstrated that there were IL-22 producing T cells in PBMCs from young children, majority of these cells were CD4 + T not CD8 + T cells with memory phenotype. Moreover, we found that Th22 subset existed in blood of healthy young children. The above results we observed in children are similar to adults [16, 17]. However, we found that the percentages of IL-22 + CD4 + T cells were significantly lower in children than adults. Previous reports observed that human Langerhans cells and mast cells induced distinct IL-22-producing CD4 + T cells [18, 19]. Existence of C. albicans or Mycobacterium tuberculosis specific IL-22 + CD4 + T cells in PBMCs of healthy adults has also been observed [16, 20]. Increased levels of frequencies of IL-22 + CD4 + T cells have been observed in patients with psoriasis, active systemic lupus erythematosus (SLE) and rheumatic arthritis (RA) [21–23]. Above results indicate that some pathogens or specific circumstances induce IL-22 + CD4 + T cell differentiation. Lower levels of IL-22 + CD4 + T cells in young children compared to adults suggests that accumulation of exposure to environmental antigens may lead to higher frequencies of IL-22 + CD4 + T cells in PBMCs.

Concurrently, we compared frequencies of Th17 from both young children and adults. As to our knowledge, this is the first report a comparison of Th17 cells from young children and adults. We found that there were significantly lower frequencies of Th17 cells from young children compared with adults. A previous study determined the capacity of naive CD4 T cells to develop into Th17 cells and found that this was inversely related to developmental age [24]. Th17 cells play a significant role in the pathogenesis of multiple inflammatory and autoimmune diseases; however, Th17 cells also contribute to maintain mucosal barriers and defend against pathogens at mucosal surfaces. The protective effects of Th17 cells against some kinds of extracellular bacterial and fungal pathogens filled a critical void in host immunity not covered by the Th1 or Th2 lineage [25–28]. Lower levels of Th17 frequencies in young children may be one reason that children are prone to infections.

Moreover, we observed frequencies of Th1, Th2 and Tc1 from young children and adults. Similar to IL-22 + CD4 + T cells and Th17 cells, frequencies of these three subsets were remarkably lower in young children than in adults. Consistent with our findings, previous studies have demonstrated that the number and percentages of Th1 and Tc1, and Th2 cells increase with age but remain significantly lower in children than levels found in adults [29–31]. Furthermore, we divided young children into 2 groups, Group A (1–3 years old) and Group B (4–6 years old). Comparison between these two groups highlighted that levels of Th1 frequencies from Group A were markedly lower than from Group B. However, there were no significant differences of levels of Th17 and IL-22 + CD4 + T cells frequencies between these two groups. This result may be explained by various speeds of accumulation among the different Th lineages as well as small sample size in this study.

As naive T cells do not generally secrete IL-22, IL-17, IFN-γ or IL-4, flow cytometric analysis of circulating levels of IL-22 + CD4 + T cells, Th17 and other T subpopulations might reflect antigen-driven T cell differentiation as the immature immune system develops. Next we determined memory phenotype CD45RO expression on CD4 + T or CD8 + T cells. The results demonstrated that both frequencies of CD4 + T and CD8 + T memory cells remained lower levels in young children than in adults. Moreover, there was significant correlation (*r* ≥ 0.6) between IL-22 + CD4 + T cells, Th17 and Th1 cell frequencies with CD4+ memory T cells respectively. Consistent with our findings, previous studies have observed that frequencies of CD4 + CD45RO+ or CD8 + CD45RO+ memory T cells increased with age [1, 2]. In addition, Th1 and Tc1 cell populations with strong correlation to CD45RO surface antigen expression was observed by a previous study [30].

## Conclusions

In summary, we demonstrated that Th22 subset existed in the peripheral blood of young children and evaluated the phenotype of this subset. Compared with adults, there were lower frequencies of IL-22 + CD4 + T cells, Th1, Th17, Th2 and Tc1 subsets in the peripheral blood of young children. Furthermore, we determined that there were lower percentages of memory CD4+ in young children than in adults which correlated with IL-22 + CD4 + T cells, Th1, and Th17 respectively. Lower frequencies of Th or Tc1 effector subsets in young children might explain in part why young children are prone to infections.

## Abbreviations

AHR, Aryl hydrocarbon receptor; BFA, Brefeldin A; FACS, fluorescence-activated cell sorting; FCM, flow cytometry; FCS, fetal calf serum; IFN-γ, interferon- gamma; IL, interleukin; PBMCs, peripheral blood mononuclear cells; PBS, phosphate buffered-saline; PMA, phorbol myristate acetate; RA, rheumatic arthritis; RORγt, retinoid acid-related orphan receptor gamma; RPMI 1640, Roswell Park Memorial Institute 1640; SLE, systemic lupus erythematosus; T-bet, T-box 21 transcription factor; Tc, cytotoxicity T cell; Th, helper T cell

## References

[1] Schatorjé EJ, Gemen EF, Driessen GJ, Leuvenink J, van Hout RW, de Vries E (2012). Paediatric reference values for the peripheral T cell compartment. Scand J Immunol.

[2] Shearer WT, Rosenblatt HM, Gelman RS, Oyomopito R, Plaeger S, Stiehm ER, Wara DW, Douglas SD, Luzuriaga K, McFarland EJ (2003). Lymphocyte subsets in healthy children from birth through 18 years of age: the Pediatric AIDS Clinical Trials Group P1009 study. J Allergy Clin Immunol.

[3] Farber DL, Yudanin NA, Restifo NP (2014). Human memory T cells: generation, compartmentalization and homeostasis. Nat Rev Immunol.

[4] Hall CB, Weinberg GA, Iwane MK, Blumkin AK, Edwards KM, Staat MA, Auinger P, Griffin MR, Poehling KA, Erdman D (2009). The burden of respiratory syncytial virus infection in young children. N Engl J Med.

[5] Hsieh C-S, Macatonia SE, Tripp CS, Wolf SF, O’Garra A, Murphy KM (1993). Development of TH1 CD4+ T cells through IL-12 produced by Listeria-induced macrophages. Science (New York, NY).

[6] Mosmann TR, Cherwinski H, Bond MW, Giedlin MA, Coffman RL (1986). Two types of murine helper T cell clone. I. Definition according to profiles of lymphokine activities and secreted proteins. J Immunol.

[7] Mosmann TR, Sad S (1996). The expanding universe of T-cell subsets: Th1, Th2 and more. Immunol Today.

[8] Harrington LE, Hatton RD, Mangan PR, Turner H, Murphy TL, Murphy KM, Weaver CT (2005). Interleukin 17–producing CD4+ effector T cells develop via a lineage distinct from the T helper type 1 and 2 lineages. Nat Immunol.

[9] Park H, Li Z, Yang XO, Chang SH, Nurieva R, Wang Y-H, Wang Y, Hood L, Zhu Z, Tian Q (2005). A distinct lineage of CD4 T cells regulates tissue inflammation by producing interleukin 17. Nat Immunol.

[10] Zhu J, Yamane H, Paul WE (2010). Differentiation of effector CD4 T cell populations. Annu Rev Immunol.

[11] Duhen T, Geiger R, Jarrossay D, Lanzavecchia A, Sallusto F (2009). Production of interleukin 22 but not interleukin 17 by a subset of human skin-homing memory T cells. Nat Immunol.

[12] Trifari S, Kaplan CD, Tran EH, Crellin NK, Spits H (2009). Identification of a human helper T cell population that has abundant production of interleukin 22 and is distinct from TH-17, TH1 and TH2 cells. Nat Immunol.

[13] Leung JM (2013). A role for IL-22 in the relationship between intestinal helminths, gut microbiota and mucosal immunity. Int J Parasitol.

[14] Sonnenberg GF, Fouser LA, Artis D (2011). Border patrol: regulation of immunity, inflammation and tissue homeostasis at barrier surfaces by IL-22. Nat Immunol.

[15] Zenewicz LA, Flavell RA (2011). Recent advances in IL-22 biology. Int Immunol.

[16] Liu Y, Yang B, Zhou M, Li L, Zhou H, Zhang J, Chen H, Wu C (2009). Memory IL‐22‐producing CD4+ T cells specific for Candida albicans are present in humans. Eur J Immunol.

[17] Shen E, Yang B, Li Y, Lin Q, Zou R, Huang H, Wu J, Zhang S, Zou J, Zhou M (2014). CsA inhibits IL-22 production by memory CD4+ T cells from patients with psoriasis. Arch Biol Sci.

[18] Fujita H, Nograles KE, Kikuchi T, Gonzalez J, Carucci JA, Krueger JG (2009). Human Langerhans cells induce distinct IL-22-producing CD4+ T cells lacking IL-17 production. Proc Natl Acad Sci.

[19] Gaudenzio N, Laurent C, Valitutti S, Espinosa E (2013). Human mast cells drive memory CD4+ T cells toward an inflammatory IL-22+ phenotype. J Allergy Clin Immunol.

[20] Zeng G, Chen CY, Huang D, Yao S, Wang RC, Chen ZW (2011). Membrane-bound IL-22 after de novo production in tuberculosis and anti-Mycobacterium tuberculosis effector function of IL-22+ CD4+ T cells. J Immunol.

[21] Zhang L, Li J-m, Liu X-g, Ma D-x, Hu N-w, Li Y-g, Li W, Hu Y, Yu S, Qu X (2011). Elevated Th22 cells correlated with Th17 cells in patients with rheumatoid arthritis. J Clin Immunol.

[22] Zhao L, Jiang Z, Jiang Y, Ma N, Wang K, Zhang Y, Feng L (2013). IL-22+ CD4+ T-cells in patients with active systemic lupus erythematosus. Exp Biol Med.

[23] Zhao L, Jiang Z, Jiang Y, Ma N, Zhang Y, Feng L, Wang K (2013). IL‐22+ CD4+ T cells in patients with rheumatoid arthritis. Int J Rheum Dis.

[24] Black A, Bhaumik S, Kirkman RL, Weaver CT, Randolph DA (2012). Developmental regulation of Th17‐cell capacity in human neonates. Eur J Immunol.

[25] Bettelli E, Korn T, Kuchroo VK (2007). Th17: the third member of the effector T cell trilogy. Curr Opin Immunol.

[26] Curtis MM, Way SS (2009). Interleukin‐17 in host defence against bacterial, mycobacterial and fungal pathogens. Immunology.

[27] Weaver CT, Harrington LE, Mangan PR, Gavrieli M, Murphy KM (2006). Th17: an effector CD4 T cell lineage with regulatory T cell ties. Immunity.

[28] Weaver CT, Hatton RD, Mangan PR, Harrington LE (2007). IL-17 family cytokines and the expanding diversity of effector T cell lineages. Annu Rev Immunol.

[29] Buck R, Cordle C, Thomas D, Winship T, Schaller J, Dugle J (2002). Longitudinal study of intracellular T cell cytokine production in infants compared to adults. Clin Exp Immunol.

[30] Chipeta J, Komada Y, Zhang X-L, Deguchi T, Sugiyama K, Azuma E, Sakurai M (1998). CD4+ and CD8+ cell cytokine profiles in neonates, older children, and adults: increasing T helper type 1 and T cytotoxic type 1 cell populations with age. Cell Immunol.

[31] Smart JM, Kemp AS (2001). Ontogeny of T‐helper 1 and T‐helper 2 cytokine production in childhood. Pediatr Allergy Immunol.

